# Distributed Transmit Power Control for Energy-Efficient Wireless-Powered Secure Communications

**DOI:** 10.3390/s21175861

**Published:** 2021-08-31

**Authors:** Kisong Lee

**Affiliations:** Department of Information and Communication Engineering, Dongguk University, Seoul 04620, Korea; kslee851105@gmail.com; Tel.: +82-2-2260-3233

**Keywords:** secure communication, energy harvesting, secrecy energy efficiency, transmit power control, distributed algorithm

## Abstract

In this study, we consider energy-efficient wireless-powered secure communications, in which *N* sets of transmitter, receiver, and energy harvesting (EH) nodes exist; each EH node is allowed only to harvest energy from the transmitted signals but is not to permitted to decode information. To maximize the sum secrecy energy efficiency (SEE) of the node sets while ensuring minimum EH requirement for each EH node, we propose a distributed transmit power control algorithm using a dual method, where each transmitter adjusts its transmit power iteratively until convergence without sharing information with the other node sets. Through simulations under various environments, we show that the proposed scheme surpasses conventional schemes in terms of the sum SEE and has significantly reduced computation time compared with the optimal scheme, which suggests the effectiveness and applicability of the proposed distributed method.

## 1. Introduction

With the rapid growth of mobile traffic and smart devices, next-generation wireless communications are expected to have increased demands for high data rates, wide coverage, and high levels of security [1,2]. At the same time, the energy consumption would inevitably increase as the functions of mobile devices become more diverse. This may cause inconvenience to users as the battery may need to be recharged or replaced more often to extend the lifetime of the network. Accordingly, several methods, such as energy-efficient protocols [3], resource allocation [4,5], and multi-antenna techniques [6,7], have been proposed to improve the energy efficiencies of networks.

In addition to energy-efficient use of resources, energy harvesting (EH) technology has emerged to solve the problem of energy shortages in wireless nodes [8,9,10,11,12]. In particular, the methods to design EH systems were proposed in [8,9], and the potential of EH technology as a promising means to enable self-sustainable operations of wireless nodes was discussed in [10,11,12]. Recent investigations on energy efficiency optimization have been also reported for wireless-powered networks, in which EH-enabled nodes exist [13,14,15,16,17]. In [13], joint optimization of user scheduling and power allocation was studied to maximize energy efficiency considering the characteristics of EH. The authors of [14] investigated a proportional fair energy efficiency method that considers energy efficiency and user fairness simultaneously. In [15,16], resource allocation strategies were proposed to improve the energy efficiency of wireless-powered cognitive radio networks (CRNs). Moreover, the authors of [17] proposed the max–min antenna selection scheme for EH-based two-way relaying and analyzed the diversity gain.

Network diversification also causes increased concerns regarding security issues between different networks because there is increased risk of eavesdropping by unlicensed users when secret keys are shared between legitimate users [1,2]. In this context, a number of studies have been conducted on physical layer security (PLS) to ensure secure communications without relying on secret keys, such as cooperative relaying [18,19] and jamming signal transmission [20]. Regarding PLS and EH simultaneously, optimal policies for EH-enabled relays [21,22] and an optimal resource allocation considering transmit power and EH ratio [23] have been proposed for wireless-powered secure communications (WPSCs). The optimal policy of a friendly jammer capable of EH has also been studied to help secure communication between licensed nodes [24].

Unlike the aforementioned studies that focus only on energy efficiency [3,4,5,6,7,13,14,15,16,17] or PLS [18,19,20,21,22,23,24], secrecy energy efficiency (SEE), which is defined as the ratio of the secrecy rate to dissipated power, was suggested to achieve a balance between energy efficiency and secure communication [25,26,27,28,29,30]. For example, resource allocations including transmit power and beamforming vectors were optimized to maximize the SEE in CRNs [25,27] and multi-antenna systems [26,28], respectively. Furthermore, the problem of SEE optimization was investigated in multi-antenna and multi-user systems with confidentiality and reliability constraints [29], and the optimal control parameters including first- and second-layer power splitting ratios, beamforming vectors, and artificial noise covariance matrix were derived to maximize the SEE in multi-antenna wireless-powered networks [30]. However, only simple scenarios without co-channel interference were considered in [25,26], and high computational complexity and information sharing among the nodes are needed to resolve the problems noted in [27,28,29,30]. Therefore, a distributed algorithm with low complexity that can be operated in real systems is required for energy-efficient WPSCs with co-channel interference. The literature survey is summarized in Table 1.

In this study, we investigate a distributed transmit power control (TPC) for energy-efficient WPCSs. The main contributions can be summarized as follows.

- We consider WPSCs where the EH nodes harvest energy from the transmitted signals but are not allowed to decode private information shared between the transmitter and receiver (Tx-Rx) pairs. Given this scenario, we formulate an optimization problem to find the optimal transmit powers of the Txs to maximize the sum SEE while guaranteeing that the amount of energy collected from each EH node is greater than a minimum required value.
- To solve this non-convex problem with low complexity, we propose a distributed TPC algorithm using dual decomposition, where each Tx determines its transmit power iteratively until convergence without sharing any information with the other node sets.
- Through performance evaluations under various environments, we show that the proposed scheme achieves a higher sum SEE than conventional schemes and remarkably reduced computation time compared with the optimal scheme.

The remainder of this paper is organized as follows. In Section 2, we provide the problem statement for the considered system model, and the distributed TPC algorithm for energy-efficient WPSCs is proposed in Section 3. In Section 4, the performance evaluations are shown through extensive simulations, and the conclusions are presented in Section 5.

## 2. System Model and Problem Statement

**Figure 1:**
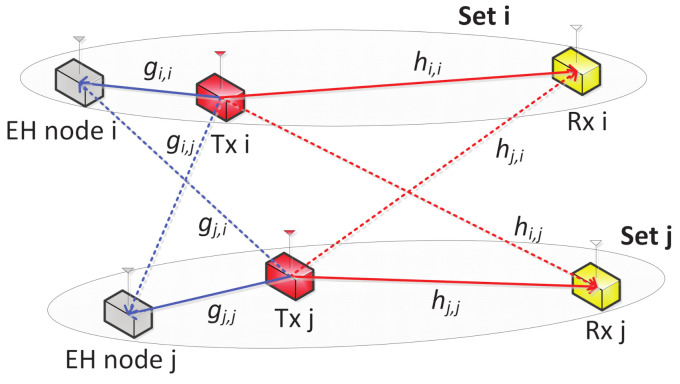
System model of WPSCs showing only two node sets for brevity.

Figure 1 shows the schematic of the WPSCs, where *N* number of Tx-Rx pairs use the same frequency band for data transmissions while *N* number of EH nodes are permitted to only collect energy from the signals sent by the Txs; all nodes are equipped with single antenna. Since the EH nodes are not allowed to interpret the information shared between their corresponding Tx-Rx pairs, they are called untrusted nodes, and each Tx is required to adjust its transmit power to maintain information confidentiality while supplying sufficient energy to guarantee the minimum EH requirement for its EH node. The EH node associated with each Tx-Rx pair is assumed to be predetermined, and the set of these nodes is denoted as N, i.e., |N|=N. The channel gain between Tx *i* and Rx *j* is denoted as $h_{i,j}$ and that between Tx *i* and EH node *j* is denoted as $g_{i,j}$, which are assumed to follow a discrete time block-fading model.

Then, the signal received at Rx *i* is represented by

(1) $$\begin{array}{c} y_{i}=\sqrt{p_{i}}h_{i,i}x_{i}+\sum_{k\in N\setminus \{i\}}\sqrt{p_{k}}h_{k,i}x_{k}+z_{i}, \end{array}$$

where $x_{i}$ denotes the normalized data symbol transmitted by Tx *i* with transmit power $p_{i}$, and $z_{i}∼\mathrm{CN}\left(0,\sigma^{2}\right)$ indicates the noise at Rx *i*.

From (1), the achievable spectral efficiency (SE) is obtained as

(2) $$\begin{array}{cc} r_{i} & =\log_{2}\left(1+\frac{p_{i}{\left|h_{i,i}\right|}^{2}}{\sigma^{2}+\sum_{k\in N\setminus \{i\}}p_{k}{\left|h_{k,i}\right|}^{2}}\right). \end{array}$$

At the same time, the signal received at EH node *i* is expressed as

(3) $$\begin{array}{c} y_{i}^{e}=\sqrt{p_{i}}g_{i,i}x_{i}+\sum_{k\in N\setminus \{i\}}\sqrt{p_{k}}g_{k,i}x_{k}+z_{i}^{e}, \end{array}$$

where $z_{i}^{e}∼\mathrm{CN}\left(0,\sigma^{2}\right)$. Considering that each EH node can harvest energy not only from the signal sent by Tx *i* but also from the signals sent by other Txs, the total harvested energy at EH node *i* is given by

(4) $$\begin{array}{cc} e_{i} & =\sum_{j\in N}\zeta_{i}p_{j}{\left|g_{j,i}\right|}^{2}, \end{array}$$

where $\zeta_{i}$ is the energy conversion efficiency. On the other hand, if EH node *i* overhears the signal sent by Tx *i* instead of harvesting energy, its achievable SE is represented by

(5) $$\begin{array}{cc} r_{i}^{e} & =\log_{2}\left(1+\frac{p_{i}{\left|g_{i,i}\right|}^{2}}{\sigma^{2}+\sum_{k\in N\setminus \{i\}}p_{k}{\left|g_{k,i}\right|}^{2}}\right). \end{array}$$

From (2) and (5), the secrecy rate of node set *i* can be defined as the rate difference between the legitimate and eavesdropping links [31] as follows:

(6) $$\begin{array}{cc} r_{i}^{s} & ={\left[r_{i}-r_{i}^{e}\right]}^{+}, \end{array}$$

where ${\left[\cdot \right]}^{+}=\max\left(0,\cdot \right)$.

Moreover, the total power consumption at node set *i* can be obtained as

(7) $$\begin{array}{cc} p_{i}^{\mathrm{CE}} & =p_{C}+p_{i}-e_{i}, \end{array}$$

where $p_{C}$ is the constant energy consumed by the circuits of each node set.

From (6) and (7), the SEE of node set *i* can be defined as the ratio of secrecy rate to total power consumption (bits/Hz/Joule), which can be formulated as follows:

(8) $$\begin{array}{cc} \eta_{i}^{s} & =\frac{r_{i}^{s}}{p_{i}^{\mathrm{CE}}}. \end{array}$$

It should be noted that this metric indicates how efficiently energy can be used to transmit secret information.

Based on these equations, we develop the optimization problem to find the optimal transmit powers of the Txs to maximize the sum SEE while guaranteeing the minimum EH requirement, $E_{\min}$, for each EH node, as follows.

(9) $$\begin{array}{cc} \max_{0⪯\vec{p}} & \;\;\;\;\sum_{i\in N}\eta_{i}^{s}\\ s.t. & \;\;\;\;e_{i}\geq E_{\min},\;\;i\in N\\ \; & \;\;\;\;p_{i}\leq P_{\max},\;\;i\in N, \end{array}$$

where $\vec{p}=\left\{p_{1},p_{2},\cdots ,p_{N}\right\}$ and $P_{\max}$ is the maximum transmit power allowed for each Tx. The problem in (9) is non-convex because of the fractional objective function and interference term; hence, the optimal solution of $\vec{p}$ cannot be derived in a closed-form. The optimal solutions can be numerically found by brute-force search, where each $p_{i}$ is quantized with *M* equally spaced values and all possible combinations are evaluated to find the optimal value. However, the channel state information (CSI) of all wireless links must be available to implement this method, and a high computational complexity of $O\;\left(M^{N}\right)$ is incurred, which increases exponentially with the number of node sets.

## 3. Distributed Transmit Power Control Algorithm

In this section, we present the distributed TPC algorithm, which can be operated with low complexity without sharing any information with the other node sets.

We decompose the original problem in (9) into *N* subproblems, which are then solved independently with low computational complexity [32]. In the subproblem, each Tx finds the transmit power to maximize its own SEE while ensuring the minimum EH requirement, which is formulated as follows:

(10) $$\begin{array}{cc} \max_{0\leq p_{i}} & \;\;\;\;\eta_{i}^{s}\\ s.t. & \;\;\;\;C1:\;e_{i}\geq E_{\min}\\ \; & \;\;\;\;C2:\;p_{i}\leq P_{\max}. \end{array}$$

With defining $x_{i}=\frac{r_{i}^{s}}{p_{i}^{\mathrm{CE}}}$, the objective function in (10) is converted from a fractional form to an equivalent subtractive form, $r_{i}^{s}-x_{i}p_{i}^{\mathrm{CE}}$, using nonlinear fractional programming [33]. Accordingly, the subproblem in (10) is reformulated as

(11) $$\begin{array}{cc} \max_{0\leq p_{i}} & \;\;\;\;r_{i}^{s}-x_{i}p_{i}^{\mathrm{CE}}\\ s.t. & \;\;\;\;C1\;\mathrm{and}\;C2. \end{array}$$

To derive the transmit power of each Tx using the dual method, we denote the Lagrangian function of (11) as follows.

(12) $$\begin{array}{cc} L(p_{i},\lambda_{i},\mu_{i})\; & =\;r_{i}^{s}\;-\;x_{i}p_{i}^{\mathrm{CE}}\;+\;\lambda_{i}\;\left(e_{i}\;-\;E_{\min}\right)\;+\;\mu_{i}\;\left(P_{\max}\;-\;p_{i}\right), \end{array}$$

where $\lambda_{i}\geq 0$ and $\mu_{i}\geq 0$ are the respective Lagrange multipliers of each constraint of (11).

The dual objective is then defined as

(13) $$\begin{array}{c} G\left(\lambda_{i},\mu_{i}\right)=\max_{\vec{p}⪰0}\;\;L\left(p_{i},\lambda_{i},\mu_{i}\right). \end{array}$$

Using (13), the dual problem can be formulated as

(14) $$\begin{array}{c} \min_{0\leq \lambda_{i},\;0\leq \mu_{i}}\;\;G\left(\lambda_{i},\mu_{i}\right). \end{array}$$

To find the suboptimal value of $p_{i}$, we build the Karush-Kuhn-Tucker (KKT) conditions with the complementary slackness, as follows.

(15) $$\begin{array}{cc} \; & \frac{\partial L(p_{i},\lambda_{i},\mu_{i})}{\partial p_{i}}\;=\;\frac{\partial r_{i}^{s}}{\partial p_{i}}\;-\;x_{i}\frac{\partial p_{i}^{\mathrm{CE}}}{\partial p_{i}}\;+\;\lambda_{i}\frac{\partial e_{i}}{\partial p_{i}}\;-\;\mu_{i}\;=\;0, \end{array}$$

(16) $$\begin{array}{cc} \; & \lambda_{i}\left(e_{i}-E_{\min}\right)=0, \end{array}$$

(17) $$\begin{array}{cc} \; & \mu_{i}\left(P_{\max}-p_{i}\right)=0, \end{array}$$

(18) $$\begin{array}{cc} \; & 0\leq p_{i}\leq P_{\max},\;\;0\leq \lambda_{i},\;\;0\leq \mu_{i}. \end{array}$$

Then, the transmit power that satisfies the KKT conditions in (15)–(18) can be derived as follows.

(19) $$\begin{array}{c} p_{i}\;=\;{\left[\frac{1}{\ln2\;\left(x_{i}(1\;-\;\zeta_{i}|g_{i,i}{|}^{2})+\mu_{i}\;-\;\lambda_{i}\zeta_{i}{\left|g_{i,i}\right|}^{2}\right)\;+\;t_{i}^{\left[s\right]}}\;-\;\frac{\Psi_{i}}{|h_{i,i}{|}^{2}}\right]}^{\;+}\;\;, \end{array}$$

where $\Psi_{i}=\sigma^{2}\;+\;\sum_{j\in N\setminus \{i\}}p_{j}{\left|h_{j,i}\right|}^{2}$ and $t_{i}^{\left[s\right]}$ is defined as

(20) $$\begin{array}{c} t_{i}^{\left[s\right]}=\frac{|g_{i,i}{|}^{2}}{\sigma^{2}+\sum_{l\in N}p_{l}{\left|g_{l,i}\right|}^{2}}. \end{array}$$

In (19), Rx *i* can easily calculate $\Psi_{i}$ by subtracting the signal power received from Tx *i* from the total received signal power. In addition, EH node *i* can measure $t_{i}^{\left[s\right]}$ readily because the denominator of (20) is the total received signal power at EH node *i*. Therefore, Tx *i* can determine its transmit power as shown in (19) by receiving information on $\Psi_{i}$ from Rx *i* and information on $t_{i}^{\left[s\right]}$ and $g_{i,i}$ from EH node *i*, respectively. It should be noted that although the EH node is the untrusted node, it should send information on $t_{i}^{\left[s\right]}$ and $g_{i,i}$ to Tx *i* to receive enough energy to meet the EH requirement for operation. Moreover, Tx *i* does not need to share any information with the other node sets for calculating $p_{i}$, thereby allowing the proposed algorithm to operate in a distributed manner.

Moreover, the Lagrange multipliers are updated using a gradient algorithm as follows.

(21) $$\begin{array}{cc} \lambda_{i}^{q+1} & ={\left[\lambda_{i}^{q}-\nu_{1}\left(e_{i}-E_{\min}\right)\right]}^{+},\\ \mu_{i}^{q+1} & ={\left[\mu_{i}^{q}-\nu_{2}\left(P_{\max}-p_{i}\right)\right]}^{+}, \end{array}$$

where $\nu_{1}$ and $\nu_{2}$ are sufficiently small step sizes for the update.

The operations of the proposed algorithm are described in Algorithm 1, where $\vec{\circ }=\left\{\circ_{1},\circ_{2},\cdots ,\circ_{N}\right\}$. Specifically, each Tx initializes the transmit power and Lagrange multipliers randomly and calculates the SEE with the initialized transmit power. Next, the Txs determine the transmit powers according to (19) and update the Lagrange multipliers according to (21) iteratively until the transmit powers converge. The Txs also update the SEE and total consumed power with the converged transmit power to assess the convergence of the SEE. This process is repeated until the SEE converges.
**Algorithm 1** Distributed transmit power control algorithm1: Initialize $\vec{p}$, $\vec{\lambda }$, and $\vec{\mu }$ randomly2: **repeat**3:  Set $\vec{x}={\vec{r}}^{s}/{\vec{p}}^{\mathrm{CE}}$4:  **repeat**5:   ${\vec{p}}_{\mathrm{old}}\leftarrow \vec{p}$6:   **for**
i=1 to *N*7:    Compute $p_{i}$ according to (19)8:    Update $\lambda_{i}$ and $\mu_{i}$ according to (21)9:   **end for**10:   $\vec{p}=\left\{p_{1},p_{2},\cdots ,p_{N}\right\}$11:  **until**
$∥\vec{p}-{\vec{p}}_{\mathrm{old}}∥<\epsilon$12:  Update ${\vec{r}}^{s}$ and ${\vec{p}}^{\mathrm{CE}}$ with $\vec{p}$13: **until**
$∥{\vec{r}}^{s}-\vec{x}{\vec{p}}^{\mathrm{CE}}∥<\delta$

Given that $\epsilon^{-2}$ iterations are needed to ensure that the norm of the gradient is less than ϵ in the worst-case scenario [34], the number of iterations required for convergence of the inner loop is $\epsilon^{-2}$. Moreover, *T* denotes the number of iterations required for convergence of the outer loop [35], and the computational complexity of the proposed algorithm is $O\;\left(TN^{2}\epsilon^{-2}\right)$, where $N^{2}$ is the number of computations required to calculate $\vec{p}$.

## 4. Simulation Results and Discussion

For the performance evaluations, the following system parameters are used as default unless stated otherwise: *N* = 3, $P_{\max}$ = $p_{C}$ = 30 dBm, $\sigma^{2}=-100$ dBm, $E_{\min}=-10$ dBm, and $\eta_{i}$ = 0.5 for i∈N. All nodes are distributed randomly over an area of 50 m × 50 m, in which the maximum distance of each signal link and EH link in the same node set is 15 m. Because EH circuits have low power sensitivity, e.g., −10∼−30 dBm for energy harvesters while −60∼−80 dBm for information receivers [12,36], the small size area is appropriate for wireless-powered networks. A simplified path loss model with a path loss exponent of 2.7 is considered for urban areas [21]. Moreover, Rayleigh fading is considered for the signal links to reflect the non-line-of-sight (nLoS) characteristics while Rician fading with a *K*-factor of 6 is considered for the EH links to reflect the line-of-sight (LoS) characteristics [12]. The following five schemes are considered for performance evaluation in terms of the sum SEE, which can be mathematically written as $E\left[\left(\prod_{i\in N}{𝟙}_{e_{i}\geq E_{\min}}\right)\cdot \sum_{i\in N}\eta_{i}^{s}\right]$. Note that a penalty is assigned by setting the sum SEE to zero when the minimum EH constraint is violated; therefore, the effects of EH violation are inherent in the sum SEE calculations.

- Optimal scheme: With the knowledge of the CSI of all wireless links, the near-optimal performance can be found using a brute-force search with M=100; however, the performance for N≥5 is found using a divide and conquer algorithm because of the extremely high computational complexity of the brute-force search.
- Proposed scheme: The transmit powers of the Txs are determined using the proposed algorithm given in Algorithm 1.
- $r^{s}$ max. scheme: The transmit powers of the Txs are determined to maximize the sum secrecy rate, $\sum_{i\in N}r_{i}^{s}$, which is found from the divide and conquer algorithm.
- Equally reduced power (ERP) scheme [37]: All Txs use the same transmit power that maximizes the sum SEE while meeting the minimum EH constraint, and the optimal value of the transmit power is found by one-dimensional exhaustive search.
- EH max. scheme: The Txs use their maximum transmit powers to maximize the total harvested energies of the EH nodes, $\sum_{i\in N}e_{i}$.
- Rand scheme: The transmit powers of the Txs are determined randomly.

**Figure 2:**
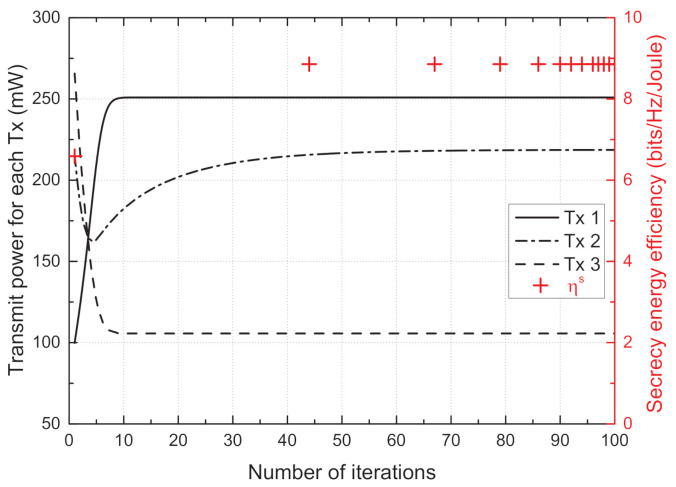
Convergence of the proposed scheme.

Figure 2 depicts the transmit power of each Tx and the sum SEE against the number of iterations, which shows the convergence of the proposed scheme. Each Tx adjusts the transmit power to maximize its own SEE, which affects the SEEs of the other node sets. Although the transmit power update at each Tx influences the other Txs, the transmit powers of all Txs converge to stationary points with iteration progression; finally, the sum SEE also converges to a value of 8.85 within 70 iterations.

**Figure 3:**
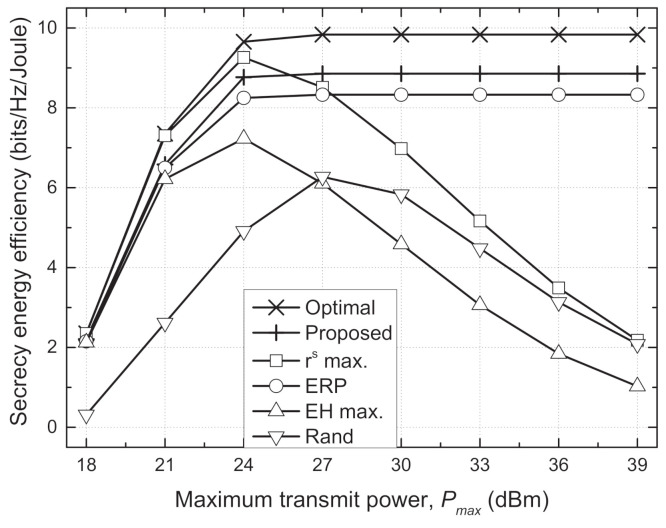
Sum secrecy energy efficiency vs. maximum transmit power ($P_{\max}$) for different schemes.

Figure 3 depicts the sum SEE versus maximum transmit power ($P_{\max}$). In the optimal, proposed, and ERP schemes, the sum SEE increases with $P_{\max}$ when $P_{\max}<27$ dBm but converges to a stationary point when $P_{\max}\geq 27$ dBm. This indicates that there is an optimal transmit power at which the maximum SEE is achieved, i.e., the use of extra transmit power beyond this value reduces the sum SEE. Therefore, these schemes do not use transmit powers of more than 27 dBm even though $P_{\max}$ increases over 27 dBm. On the other hand, the $r^{s}$ max. and EH max. schemes use more transmit power to maximize the sum secrecy rate and total harvested energy, respectively, as $P_{\max}$ increases. However, the excessive use of transmit power exceeding 27 dBm causes inefficient energy consumption rather than improvement of the secrecy rate, so the sum SEE degrades seriously when $P_{\max}$ is greater than 27 dBm. The sum SEE of the rand scheme also decreases as $P_{\max}$ increases because it uses more transmit power without the adaptive TPC strategy.

**Figure 4:**
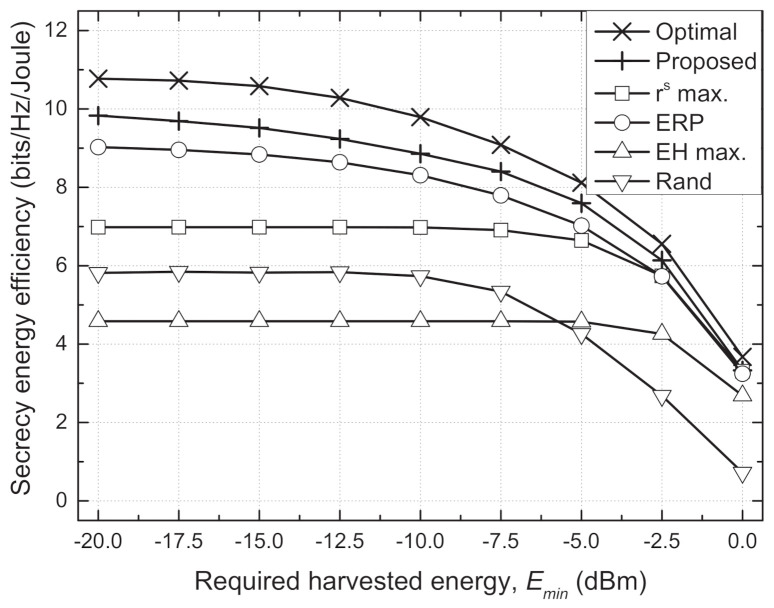
Sum secrecy energy efficiency vs. required harvested energy ($E_{\min}$) for different schemes.

**Figure 5:**
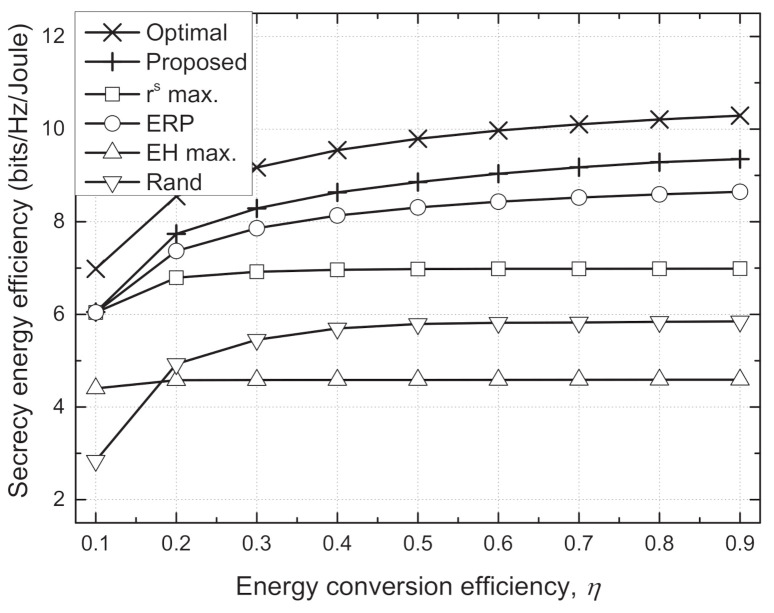
Sum secrecy energy efficiency vs. energy conversion efficiency (η) for different schemes.

Figure 4 and Figure 5 depict the sum SEE versus required harvested energy ($E_{\min}$) and energy conversion efficiency (η), respectively. In an environment where it is difficult to satisfy the EH requirements, i.e., larger $E_{\min}$ and smaller η, the Txs should use the additional transmit power inefficiently to satisfy the EH requirements. For some channel realizations, e.g., deep fading, the EH requirements cannot be inherently guaranteed at extremely large $E_{\min}$ or small η. Then, this causes serious degradation in the sum SEE because it is set to zero when the minimum EH constraint is violated. Hence, the sum SEEs of all schemes decrease as $E_{\min}$ increases or η decreases, especially when $E_{\min}\geq -5$ dBm and η≤0.3. However, we can see that the proposed scheme shows a trend similar to the optimal scheme and outperforms the conventional schemes. In particular, the performance gain of the proposed scheme compared to the conventional schemes is greater in an environment that favorably satisfies the EH requirements, where the adaptive TPC strategy can operate more effectively.

**Figure 6:**
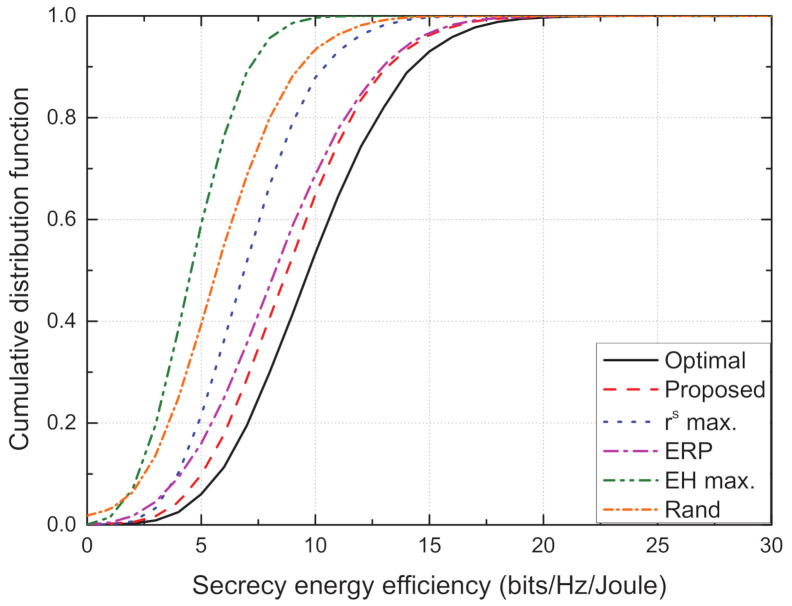
Cumulative distribution function vs. sum secrecy energy efficiency for different schemes.

Figure 6 depicts the cumulative distribution function (CDF) versus sum SEE. It is observed that the CDF of the proposed scheme is closest to that of the optimal scheme, thus confirming the effectiveness of the proposed TPC strategy. Given that high values of SEE are more distributed in the CDFs of the optimal and proposed schemes, rather than those of the conventional schemes, we note that the effective TPC is important for improving the SEE.

**Figure 7:**
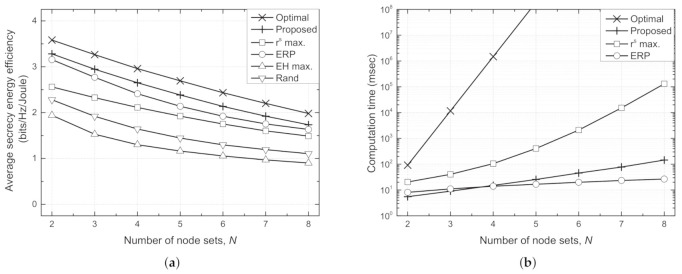
Performance comparison against number of node sets (*N*) for different schemes. (**a**) Average secrecy energy efficiency per each node set vs. *N*. (**b**) Computation time vs. *N*.

Figure 7 depicts the average SEE for each node set ($\frac{\sum_{i\in N}\eta_{i}^{s}}{N}$) and the computation time against number of node sets (*N*). It should be noted that the computation time of the EH max. and random schemes are omitted in subfigure (b) because they do not perform any computations to determine the transmit powers. As *N* increases, the Txs experience severe interference with each other. As a result, the improvement in the secrecy rate is insignificant compared to the transmit power used, which in turn deteriorates the average SEE with increasing *N*. However, the proposed scheme achieves a higher SEE than the conventional schemes by coping with the interference and reduces the computation time significantly compared with the optimal and $r^{s}$ max. schemes. Although the ERP scheme can achieve the lowest computation time for a large number of *N* because the optimal transmit power can be found by one-dimensional search even as *N* increases, the proposed scheme achieves higher sum SEE than the ERP scheme, which verifies the effectiveness of the proposed distributed method in term of energy efficiency and secure communication.

Thus, the simulation results show that the proposed scheme has a slight difference in performance from the optimal scheme of about 10% because of its distributed nature; however, it is confirmed that the proposed scheme improves the sum SEE using the effective TPC strategy compared with the conventional schemes while significantly reducing computation time.

## 5. Conclusions

This study involves investigation of a distributed TPC algorithm for energy-efficient WPSCs, in which the transmit powers of the Txs are optimized to maximize their own SEEs while guaranteeing the minimum EH requirements for the corresponding EH nodes. Specifically, we analytically derived the closed-form equation for the transmit power, and proposed an iterative algorithm using a dual method, that can be operated in a distributed manner without sharing information with the other node sets. The simulation results confirm that the proposed scheme can achieve a higher sum SEE than existing schemes by adjusting the transmit power with respect to secrecy rate and energy efficiency; moreover, the computation time is significantly reduced compared with the optimal scheme. It is expected that our solution will be used to solve the complex TPC problems of WPSCs in a simple distributed manner. Interesting topics for future work include a deep learning-based distributed TPC algorithm for improving the performance of WPSCs.

## Figures and Tables

**Table 1:** Literature Survey.

Literature	Metric	Research Issue	Limitation
[3]	EE	Protocol	Did not consider WPSCs.
[4,5]	EE	Resource allocation	Did not consider WPSCs.
[6,7]	EE	Multi-antenna tech.	Did not consider WPSCs.
[8,9]	EH capability	EH system design	Did not consider WPSCs.
[10,11,12]	Possibility of EH	Applicability of EH to netw.	Did not consider WPSCs.
[13]	EE	Resource allocation	Did not consider PLS.
[14]	Proportional fair EE	Resource allocation	Did not consider PLS.
[15,16]	EE	Resource allocation	Did not consider PLS.
[17]	EH power	Antenna selection	Did not consider PLS.
[18,19]	Secure commun.	Cooperative relaying	Did not consider EH.
[20]	Secure commun.	Jamming signal trans.	Did not consider EH.
[21,22]	Secure commun.	Optimal relaying policy	Did not consider EH.
[23]	Secure commun.	Resource allocation	Did not consider EH.
[24]	Secure commun.	Policy of a friendly jammer	Did not consider EH.
[25,26]	SEE	Resource allocation	Simple scenarios w/o interf.
[27,28,29,30]	SEE	Resource allocation	Centralized approach.

## Data Availability

Not applicable.
